# The Clinical Characteristics and Direct Medical Cost of Influenza in Hospitalized Children: A Five-Year Retrospective Study in Suzhou, China

**DOI:** 10.1371/journal.pone.0044391

**Published:** 2012-09-05

**Authors:** Tao Zhang, Qiuli Zhu, Xuelan Zhang, Yunfang Ding, Mark Steinhoff, Steven Black, Genming Zhao

**Affiliations:** 1 Department of Epidemiology, School of Public Health, Fudan University Key Laboratory of Public Health Safety, Ministry of Education, Shanghai People’s Republic of China; 2 Suzhou Children’s Hospital, Suzhou University, Suzhou, People’s Republic of China; 3 Center for Global Health, Cincinnati Children’s Hospital, Cincinnati, Ohio, United States of America; The University of Hong Kong, Hong Kong

## Abstract

**Background:**

There have been few studies on children hospitalized with influenza published from mainland China. We performed a retrospective review of medical charts to describe the epidemiology, clinical features and direct medical cost of laboratory-proven influenza hospitalized children in Suzhou, China.

**Methods:**

Retrospective study on children with documented influenza infection hospitalized at Suzhou Children Hospital during 2005–2009 was conducted using a structured chart review instrument.

**Results:**

A total of 480 children were positive by immuno-fluorescent assay for influenza during 2005–2009. The hospitalizations for influenza occurred in 8–12 months of the year, most commonly in the winter with a second late summer peak (August-September). Influenza A accounted for 86.3%, and of these 286 (59.6%) were male, and 87.2% were <5 years of age. The median length of hospital stay was 7 days. Fever was the most common symptom, occurring in 398 (82.9%) children. There were 394 (82.1%) children with pneumonia and 70.2% of these hospitalized children had radiographic evidence of a pulmonary infiltrate. One hundred and twelve children (23.3%) required oxygen treatments and 13 (2.7%) were transferred to the ICU. Multivariable logistic regression showed that compared with the ≤6 months children, those aged >60 months old had shorter hospital stay (OR = 0.45); children with oxygen treatment tended to have longer hospital stays than those without oxygen treatment (OR = 2.14). The mean cost of each influenza-related hospitalization was US$ 624 (US$ 1323 for children referred to ICU and US$ 617 for those cared for on the wards). High risk children had higher total cost than low-risk patients.

**Conclusion:**

Compared to other countries, in Suzhou, children hospitalized with influenza have longer hospital stay and higher percentage of pneumonia. The direct medical cost is high relative to family income. Effective strategies of influenza immunization of young children in China may be beneficial in addressing this disease burden.

## Introduction

Seasonal epidemics of influenza are responsible for respiratory diseases in children each year, resulting substantial morbidity, mortality and increased burden on health utilization, but this has tended to be under-recognized, especially in the Asian region [1], [2]. Various studies have indicated that young children are at high risk for serious illness and hospitalization from influenza virus infection [3]–[4]. In the United States, 80% of influenza-associated hospitalization in children occur in children<5 years of age and more than half of influenza mortality among children also occurs in this age group. A study using an excess-hospitalization analysis framework showed that admission rates in Hong Kong for influenza were 3 to 10 times as high as those reported for children in the United States [5]. Additionally, several studies [6]–[7] have shown that young children are important in the spread of community influenza since they often introduce the infection to their families, disseminate infection through schools to the broader community with accumulating evidences demonstrating that immunization of young children can reduce disease rates in unimmunized members of the local community [8]. In 2009, the American Advisory Committee on Immunization Practices (ACIP) recommended that annual influenza vaccination be administrated to all children 6 months -18years [9]. In contrast, universal influenza vaccination is not yet recommended for children in China; this is partly due to lack of data regarding the burden of laboratory-confirmed influenza in children.

Influenza typically presents as an upper respiratory tract infection and less commonly as serious low respiratory tract infection. Pneumonia, acute otitis media, bronchiolitis, asthma, febrile convulsion and encephalopathy are common clinical illnesses caused by influenza [10]. Although hospitalization represents only a minority of influenza cases in children, it results in a considerable economic and social burden. The epidemiology and burden of disease of influenza, especially in the 87 million children less than five years of age in China has not been described. Importantly, influenza vaccination is not routinely recommended in China and immunization of children is uncommon.

In order to further the understanding of the clinical characteristics and direct medical cost of hospitalized influenza in children from China, a comprehensive retrospective analysis of available laboratory and hospital admission data was undertaken at the Suzhou Children Hospital (SCH) in Jiangsu province in China.

## Methods

### Study Site

This retrospective study was conducted in children admitted to SCH. SCH is the single tertiary children’s hospital serving almost all young children in Suzhou. Suzhou is the largest city in Jiangsu Province in eastern China, having a population of approximately 12 million people, half of whom are migrant population. The <15 year old population of Suzhou residents in 2010 was 642,416, the migrant population of <15 years was 489,558. Suzhou is located 60 km north of Shanghai, and has a typical subtropical monsoon climate with an annual average temperature of 17.4°C and annual precipitation of 1175.7 mm.

**Table 1:** Population profile and primary clinical characteristics of influenza positive children hospitalized in Suzhou Children Hospital from 2005 to 2009 (%).

		Influenza A (n = 242)	Influenza B (n = 66)	pH1N1 (n = 172)	*χ^2^*	*P* value
Gender	Male	145(59.9)	39(59.1)	102(59.3)	0.02	0.99
	Female	97(41.8)	27(40.9)	70(40.7)		
Age	≤6 m	51(21.1)	12(18.2)	67(39.0)	34.0	<0.001
	7 m-	98(40.5)	18(27.3)	35(20.3)		
	25 m-	68(28.1)	21(31.8)	48(27.9)		
	>60 m	25(10.3)	15(22.7)	22(12.8)		
Year	2005	47(19.4)	7(10.6)	-	371.0	<0.001
	2006	70(28.9)	26(39.4)	-		
	2007	48(19.8)	9(13.6)	-		
	2008	53(21.9)	16(24.2)	-		
	2009	24(9.9)	8(12.1)	172(100.0)		
Location^a^	Suzhou Township	149(61.6)	44(66.7)	111(64.5)	3.6	0.46
	Neighboring Counties	76(31.4)	15(22.7)	52(30.2)		
	Others	17(7.0)	7(10.6)	9(5.2)		
Symptoms	Fever	204(84.3)	59(89.4)	135(78.5)	4.7	0.10
	Respiratory distress	10(4.1)	0(0.0)	4(2.3)	3.5	0.18
	X-ray abnormal	164(67.8)	47(71.2)	126(73.3)	1.5	0.48
Coexisting condition	Asthma	29(12.0)	6(9.1)	9(5.2)	5.5	0.06
	Cardiovascular disease	7(2.9)	0(0.0)	2(1.2)	3.1	0.21
	Liver diseases	1(0.4)	1(1.5)	5(2.9)	4.4	0.11
	Others	4(1.7)	1(1.5)	5(2.9)	0.9	0.64
Complications	Pneumonia	190(78.5)	54(81.8)	150(87.2)	5.2	0.08
	Sinusitis	25(10.3)	5(7.6)	18(10.5)	0.5	0.78
	septicemia	3(1.2)	0(0.0)	2(1.2)	-	-
	Febrile convulsions	5(2.1)	3(4.5)	10(5.8)	4.0	0.13
	Diarrhea	13(5.4)	2(3.0)	15(8.7)	3.3	0.19
	Impaired Liver function	5(2.1)	0(0.0)	7(4.1)	3.6	0.16
Therapeutics	oxygen Treatment	38(9.1)	8(4.5)	66(8.1)	34.3	<0.001
	Refer to ICU	6(2.5)	1(1.5)	6(3.5)	0.8	0.67
Condition at discharge	Recover	33(13.6)	15(22.7)	3(1.7)	18.4	0.001
	Improved	207(85.5)	51(77.3)	168(97.7)		
	Uncured	2(0.8)	0(0.0)	1(0.6)		
Hospitalization duration (days)		7.0(6.0–9.0)	7.0(6.0–9.0)	8.0(6.0–9.0)	0.5	0.763
Total hospitalization cost (US$)		591(457–789)	557(436–728)	696(509–905)	17.9	<0.001

Note :^a^township means the urban districts in the middle of Suzhou city; neighboring counties means the suburban counties surround the urban districts; others are outside of Suzhou city.

### Study Subjects

From February 2005 to December 2009, the children who were hospitalized at SCH with acute respiratory infection had nasal aspirate specimens collected within 24 hours after admission, and the specimens were sent to the laboratory to determine influenza virus infection by direct immuno-fluorescent testing (D3 Ultra DFA respiratory virus screening and identification kit, Athens, Ohio, USA) [11]. All children who were positive for influenza were included in this study. The immuno-fluorescent test we used detects influenza A and influenza B virus, but does not specifically identify the 2009 pandemic H1N1 (pH1N1) strain. Since surveillance conducted using pcr by the China CDC branch in Suzhou revealed that influenza virus circulation in the period between August 1^st^, 2009 and March 31^st^, 2010 was almost exclusively pH1N1 [12], we assumed that all the influenza A cases identified between this period were pH1N1.

**Figure 1:**
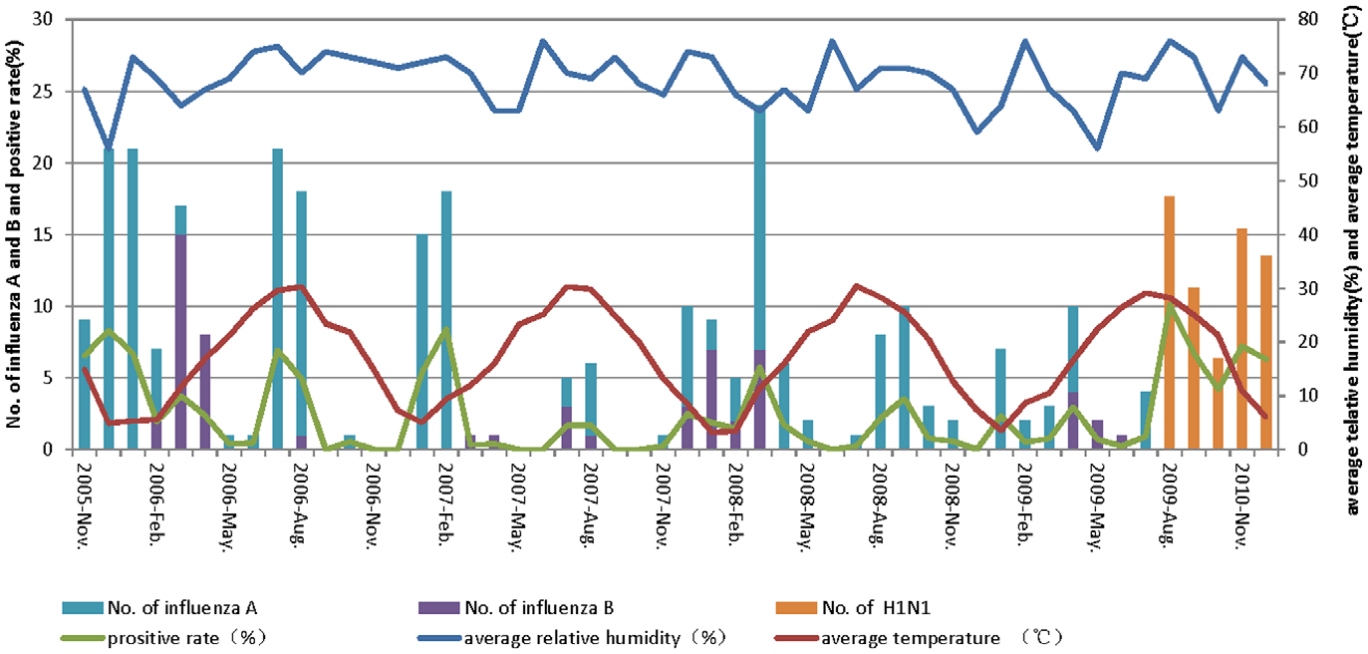
Monthly distribution of number of influenza cases, positive rate, precipitation and mean temperature in Suzhou from Nov 2005 to Dec 2009.

### Data Collection

Data on the demographic, clinical symptoms and complications, coexisting conditions (including chronic pulmonary, cardiovascular, renal, hepatic, neurological/neuromuscular, hematologic, and metabolic disorders.), therapy, and laboratory findings were abstracted from medical charts by a structured medical record review. The cost of hospitalization was obtained from the hospital billing data system which contains detailed line-item charges for all diagnostic tests, therapeutics, supplies and room fees. We used an exchange rate of 1 USD for 6.3 RMB to convert RMB costs to US dollars. Meteorological data were provided by the Suzhou Meteorological Bureau.

**Table 2:** Clinical characteristics by age group of the influenza positive children hospitalized in Suzhou Children Hospital from 2005 to 2009.

	≤6 m (n = 130)		7 m- (n = 151)		25 m- (n = 137)		>60 m (n = 62)		Total (n = 480)	
	n	%	N	%	n	%	N	%	n	%
Symptoms										
Fever*	83	63.8	128	84.8	128	93.4	59	95.2	398	82.9
Respiratory distress*	7	5.4	6	4.0	0	0.0	1	1.6	14	2.9
X-ray abnormal*	103	79.2	103	68.2	85	62.0	46	74.2	337	70.2
Oxygen saturation*										
Test no.	27	20.8	14	9.3	10	7.3	5	8.1	56	11.7
<95%	9	33.3	4	28.6	2	20.0	0	0.0	15	26.8
CRP test no.	119	91.5	134	88.7	118	86.1	49	79.0	420	87.5
CRP≥8 mg/L*	12	10.1	36	26.9	24	20.3	16	32.7	88	21.0
ACIP indication for influenza vaccine	15	11.5	21	13.9	24	17.5	10	16.1	70	14.6
Complications										
Pneumonia*	123	94.6	128	84.8	99	73.3	44	72.1	394	82.1
Sinusitis*	3	2.3	12	7.9	17	12.4	16	25.8	48	10.0
septicemia	3	2.3	1	0.7	1	0.7	0	0.0	5	1.0
Febrile convulsions*	0	0.0	5	3.3	11	8.0	2	3.2	18	3.8
Diarrhea*	14	10.8	13	8.6	2	1.5	1	1.6	30	6.2
Abnormal liver function*	9	6.9	3	2.0	0	0.0	0	0.0	12	2.5
oxygen Tx	32	24.6	35	23.2	29	21.2	12	19.4	112	23.3
Refer to ICU	6	4.6	4	2.6	1	0.7	2	3.2	13	2.7
Length of hospital stay(day)										
Median	8.0		8.0		7.0		7.0		7.0	
IQR^a^	6.0–9.0		6.0–10.0		6.0–9.0		5.0–8.0		6.0–9.0	

Notes: *p<0.05.

a: IQR, inter-quartile range.

The criteria for diagnosing pneumonia was a clinical diagnosis of pneumonia by the treating physicians according to the syndromes and chest x-ray evidence as recorded in chart. Respiratory distress was diagnosed if there were 2 or more of the following items were present: tachypnea (<2 m, RR>60/min; 2–12 m, RR>50/min; >12 m, RR>40/min); irritability; moaning while breathing; chest wall inspiratory depression; nasal flaring; moist rales or tubular breath sounds; abnormal chest X-ray; Decreased O_2_ saturation.

**Table 3:** Analysis of potential risk factors for the length of hospitalization of influenza positive children in Suzhou Children Hospital.

Factors	N(%)	COR (95%CI)	AOR (95%CI)	*P* value
Age groups				
≤6 m	130(27.1)	1.00	1.00	
7 m-	151(31.5)	1.27(0.79–2.04)	1.45(0.88–2.41)	0.148
25 m-	137(28.5)	0.75(0.47–1.22)	0.91(0.54–1.55)	0.731
>60 m	62(12.9)	0.40(0.21–0.76)	0.45(0.23–0.90)	0.024
Gender				
Female	194(40.4)	1.00	1.00	
Male	286(59.6)	1.27(0.88–1.82)	1.17(0.80–1.71)	0.425
Insurance				
No	435(90.6)	1.00	1.00	
Yes	45(9.4)	0.89(0.48–1.64)	0.91(0.46–1.80)	0.779
Fever				
No	82(17.1)	1.00	1.00	
Yes	398(82.9)	0.57(0.35–0.92)	0.71(0.42–1.21)	0.203
Respiratory distress				
No	466(97.1)	1.00	1.00	
Yes	14(2.9)	2.63(0.81–8.51)	1.22(0.35–4.30)	0.759
oxygen Tx				
No	368(76.7)	1.00	1.00	
Yes	112(23.3)	2.22(1.43–3.43)	2.14(1.32–3.48)	0.002
X-ray abnormal				
No	143(29.8)	1.00	1.00	
Yes	337(70.2)	1.47(0.99–2.18)	1.40(0.92–2.13)	0.113
ACIP indication for influenza vaccine				
No	410(85.4)	1.00	1.00	
Yes	70(14.6)	1.26(0.76–2.09)	1.22(0.72–2.08)	0.461
CRP				
<8 mg/L	332(79.0)	1.00	1.00	
≥8 mg/L	88(21.0)	0.98(0.61–1.55)	0.93(0.57–1.53)	0.784

Notes: CRP, C reaction protein; COR, crude odds ratio; AOR, adjusted odds ratio.

### Statistical Analysis

Continuous variables are presented as the mean ± SD (standard deviation) or as the median with inter-quartile ranges (IQR). Categorical variables were presented as numbers and percentages. Univariate and multivariate analysis were used to explore potential risk factors for length of hospitalization for influenza-positive children. Age, gender, insurance status, fever, oxygen treatment, presence of an ACIP indication for influenza vaccine, chest x-ray abnormal, and C reaction protein (CRP) level were entered into the logistic regression model, and the length of hospitalization was recoded as a binary variable with the median as cut point. Odds ratios (OR) and 95% confidence intervals (CI) were calculated for binomial variables. *P*-values were calculated using the chi-square test or Fisher’s exact test for categorical variables as appropriate. All tests were two-tailed and *P*-values less than 0.05 were considered statistically significant. All analysis was performed with SAS version 9.0.

**Table 4:** Influenza-related hospitalization costs on the wards and in the ICU from Suzhou, China (USD).

	Wards (n = 467)		ICU (n = 13)		Total (n = 480)	
	median	*IQR*	median	*IQR*	median	*IQR*
Diagnostics	156	117–199	217	184–289	157	119–200
Routine examination	139	102–171	184	178–213	140	103–174
Radiology test	10	6–24	–	–	10	6–24
Others	29	29–39	50	33–80	29	29–39
Therapeutics	265	177–359	617	433–804	270	178–365
Western medicine	257	172–354	614	407–785	260	173–359
Traditional Chinese medicine	8	4–12	10	8–10	8	4–12
oxygen Tx	0	0–2	22	4–35	0	0–7
Blood products	0	0–34	19	6–32	3	0–32
Procedures	–	–	24	12–128	24	12–128
Room costs and supplies	113	84–141	316	220–433	113	85–143
Nursing	17	11–25	37	22–74	17	11–25
Room	28	17–40	33	17–61	28	17–43
Accompanying Person	9	6–12	10	9–13	9	6–12
Supplies	54	30–75	208	137–312	54	30–75
Physician services	71	46–103	124	103–166	72	47–105
Total	615	479–806	1323	1123–1585	624	480–828

Notes: (1) The exchange rate is 1 USD equal 6.3 RMB.

(2) Diagnostics including: routine examination (blood, urine, stool and blood biochemical examination); radiology tests (X-ray, CT, MRI, et al.); and other diagnostics (B-ultrasound, color Doppler ultrasound, gastric function testing, bronchoscopy, et al.).

(3) Therapeutics including: medicine, oxygen, blood, procedures (tracheostomy etc.) et al.

(4) Room costs and supplies including: nursing, room fee, and other supplies.

**Table 5:** Cost of hospitalization for the influenza infection children in Suzhou, China(USD).

	N(%)	Hospitalization Costs median (IQR)	*Z*	*P* value
Age			1.94	0.586
≤6 m	130(27.1)	591(453–790)		
7 m-	151(31.5)	633(479–886)		
25 m-	137(28.5)	630(494–825)		
>60 m	62(12.9)	628(455–849)		
Gender			2.07	0.039
Female	184(38.3)	585(463–788)		
Male	286(59.6)	644(481–857)		
Insurance			2.07	0.039
No	435(90.6)	620(477–805)		
Yes	45(9.4)	721(529–926)		
Fever			1.41	0.160
No	82(17.1)	652(509–845)		
Yes	398(82.9)	615(479–816)		
oxygen Tx			10.08	<0.001
No	368(76.7)	559(445–712)		
Yes	3112(23.3)	892(715–1093)		
X-ray abnormal			1.82	0.069
No	143(29.8)	587(477–788)		
Yes	337(70.2)	639(480–862)		
ACIP indication for influenza vaccine			3.21	0.001
No	410(85.4)	615(477–799)		
Yes	70(14.6)	716(547–1009)		
CRP level			0.92	0.358
<8 mg/L	332(79.0)	629(480–848)		
≥8 mg/L	88(21.0)	662(488–880)		

Note:kruskal-wallis test, wilcoxon test.

The exchange rate is 1 USD equal 6.3 RMB.

### Ethics statement

As a retrospective study on medical record data with no patients contact and no collection of personal data, this study was exempt from obtaining informed consent. The study was reviewed and approved by the Institute Review Board in the School of Public Health at Fudan University which is registered with the Office for Human Research protections and has a US Federal Wide Assurance (approval no. IRB #2010-01-0198) and the IRB of Cincinnati Children’s Hospital.

## Results

From February 2005 to December 2009, there were 480 children hospitalized at SCH with confirmed influenza infection. The youngest child was a 7 day old infant and the oldest was a 13-year old, with the median age being 2.29 years, and 87.2% of children being <5 years of age. Importantly, there were 130 children (27.1%) less than six months old. Overall, 286 (59.6%) children were male, and 63.3% of them are from the township of Suzhou city. (Table 1).

Of the 480 children infected with influenza, 414 (86.3%) were positive for influenza A and 66 (13.8%) for influenza B. Of the 414 influenza A positive cases, 242 were during seasonal influenza period, and 172 were during the period of pandemic H1N1 circulation. The median age of influenza A positive children was 16 month (IOR: 5–39 month), which was much younger than that of influenza B positive children of 33 month (IQR: 8–54 month) (p<0.05). There was a tendency for children infected with influenza A (and pH1N1) have more respiratory distress, more oxygen need and to be referred to the ICU, but these differences were not statistical significant. (p>0.05 for all) (Table 1).

A seasonal distribution of influenza children was observed during the four consecutive seasons from November 2005 to October 2009. Hospitalizations for influenza occurred year round, predominating in the winter and early spring (November - April) with a second late summer peak (July - September). The number of influenza-positive children and the positive rate were highest during the winter peak period except during the 2008–2009 season. For example, the monthly influenza positive rates were 8.3% in December 2005; 6.7% in January 2006; 5.4% in January and 8.4% in February 2007. And the peak time lagged to March in 2008 (5.7%). During the pandemic, the positivity rate increased to a peak of 10.1% in August 2009. The proportions of detected influenza A and B varied in each year (χ^2^ = 36.23, p<0.001). Usually, influenza A was the most frequent detected virus all year round. In the late winter of 2006 and 2008, influenza B became the dominant virus in Suzhou city. Pandemic A/H1N1 circulated from August-December 2009 as assessed by virologic surveillance by the China CDC in Suzhou using pH1N1 pcr testing. (Figure 1).

From 2005 to 2009, the average temperature was highest between June to August, with the highest temperature 30.4°C in July 2008; and usually it is coldest from December- to February, with the lowest temperature 4.1°C in February, 2005. The relative humidity varied between 56–76%. Both temperature and relative humidity were lowest during the high incidence period whereas they were highest during the second circulation period in the four consecutive seasons. (Figure 1).

Only 45(9.3%) of all the 480 influenza positive children had medical insurance in Suzhou, therefore the cost of these hospitalizations represented a substantial burden to most parents. There were 70 children with ACIP designated underlying medical conditions for influenza vaccine, and 36 (51.4%) of them were ≤2 years old, 60 (85.7%) were less than 5 years old. The proportion of ACIP indications for influenza vaccine tended to be higher in the children of ≥2 years old (Table 2). Of these children with ACIP high risk conditions, there were 62(15.0%) children infected with influenza A and 8(12.1%) children infected with influenza B. Chronic pulmonary disease–usually moderate or severe asthma (44 children or 62.9%) –was the most common underlying medical condition identified,followed by cardiovascular disease in 9 children (12.9%). There were 17 (3.5%) children who had been born prematurely.

Fever was the most frequent symptoms in the influenza positive children, occurring in 398 (82.9%) children. In total, 70.2% (339/480) children had an abnormality identified on chest X-ray. Of these, 72(21.4%) had increased lung markings, 3(0.9%) had small infiltrates, 144(42.7%) with larger infiltrates, and 118 (35.0%) had diffuse infiltrates. Overall, the most frequent complications were pneumonia (82.1%), followed by Sinusitis (9.4%), then diarrhea (6.2%), febrile convulsions (3.8%), followed by 12(2.5%) children with laboratory evidence of hepatic injury and 5 (1.0%) children with septicemia. (Table 2).

One hundred and twelve children (23.3%) received oxygen of which 73 (65% of them) received oxygen only as part of aerosol therapy. Thirty-two (28.6%) were younger than 6 months, 35 (31.3%) were 7 to 24 months old, and 41 (36.6%) were over 24 months of age. The percentage requiring oxygen treatment decreased with age. Overall, the average duration of oxygen treatment was one hour. The children infected with influenza A or pH1N1 tended to be more likely to need oxygen treatment as compared to those infected with influenza B virus (P>0.05). Thirteen children (2.7%) were referred to the ICU with a median stay of 11.0 days (IQR: 9.0–12.0 days). Twelve of them were infected with influenza A and 6 occurred during the pandemic period. The median length of hospital stay of all influenza positive children was 7.0 days. (Table 2).

For the six children who were influenza A positive and admitted to the ICU, diagnoses included: pneumonia with laryngeal obstruction (2), pneumonia (2), severe pneumonia (1), and pneumonia with encephalitis (1). During the pandemic period, six children were hospitalized in the ICU with: pneumonia (1), neonatal pneumonia and cortical dysplasia (1), neonatal pneumonia (1), eczema and H1N1 infection (1), severe pneumonia with SRDS (1), and bronchial pneumonia and respiratory failure (1).

Among the 70 children (14.6%) with ACIP indication for influenza vaccine, only 3 (4.3%) children developed severe complications–2(2.9%) children developed respiratory or heart failure and 1(1.4%) had evidence of hepatic injury as evidenced by abnormal ALT or AST. While among the 410(85.4%) children without ACIP indication for influenza vaccine, there were 31 children (7.6%) who developed a severe complication: 5(1.2%) had febrile convulsions, 7(1.7%) had respiratory or heart failure, 9(2.2%) had myocardial damage and 10(2.4%) had evidence of hepatic injury.

Univariate analysis revealed that the children >60 months old has significantly shorter length of hospitalization than those ≤6 months old (COR = 0.40), and those with oxygen treatment had significantly longer hospital stay than those without this treatment (COR = 2.63). Controlling for other variables using multivariable logistic regression, revealed that compared with the ≤6 months children, those aged >60 months old had shorter stays (AOR = 0.45); children with oxygen treatment tended to have longer hospital stays than those without this treatment (AOR = 2.14). (Table 3).

Overall, contributors to the hospitalization costs consisted of therapeutics (44%), diagnostics (25%), room costs and supplies (19%) and physician services (12%). The lowest total hospitalization cost observed in these children was US$117, highest was US$2489 and the median was US$624. The costs for those referred to ICU was US$1323 (IQR: 1123–1585), higher than that of children cared for on the wards US$615 (IQR: 479–806) (Z = 4.86, p<0.001) (Table 4).The costs during pandemic influenza period was US$696 (IQR: 509–905), higher than that of children from seasonal influenza period US$587(IQR: 456–765) (Z = 4.18, p<0.001) (Table 1). Hospitalization costs of those who had insurance, high risk factors (ACIP indications for influenza vaccine), oxygen treatments were higher than others. (Table 5).

## Discussion

This retrospective study describes the clinical characteristics and direct medical costs of the influenza positive children hospitalized at SCH from2005 to 2009. We undertook this study because the social structure and medical system in China are unique and there have been no prior evaluations of the medical costs of influenza in children in China. By national policy, almost all families in China have only one child and are highly focused on the care and wellbeing of that child. Currently, only about 9.3% of families have health insurance coverage for their children and hence must pay for the total cost of the hospitalization themselves. Both of these factors would tend to influence medical utilization in China.

Influenza-associated hospitalization in Suzhou has two distinct annual peaks. This perennial pattern is also reported by nearby Zhejiang Province in the southern China [13]. However, influenza occurs year round in tropical areas and has only one peak in the winter of temperate areas [14]–[15]. The difference may influence the timing of future influenza vaccine campaigns in this area.

As reported previously [16]–[18], temperature and humidity have an effect on influenza virus activity. Fang et al. reported [17] lower temperature and lower relative humidity were facilitated influenza virus’ transmission. In our study, both higher temperature and lower temperature were associated with influenza virus activity, although this was not statistically significant. In contrast to other studies, humidity did not effect influenza virus activity. To confirm these findings, further prospective study is needed.

Of the total of 480 influenza positive hospitalized children in our study, 86.4% were under 5 years old and 28.5% were ≤6 months old. Potentially the risk of illness in children ≤6 months of age could be prevented by maternal immunization, but influenza vaccine is not approved in China for this population [19]. In addition, we note that this age group was also the most commonly hospitalized in the ICU (6 out of the 13 children referred to ICU were ≤6 m). The elevated risk observed in this age group supports the importance of vaccination of caregivers, siblings, and other family members.

It has been reported that acute otitis media (AOM) is the most common complication for influenza-attributed hospitalizations [1], [20]–[21]. However, there were only 4 (0.8%) children diagnosed as AOM in our study perhaps reflecting the low rate of routine otoscopy use in China. A total of 48 (10.0%) children were diagnosed with sinusitis in this study. In addition, the ICU admission rate (2.7%) was much lower than reported rates for other countries with 15% of the children requiring for ICU admission in the USA [22] and 12% in Canada [23].

In our study, more than 80% of the children with laboratory-proven influenza were previously healthy whereas 14.6% had an ACIP indication for vaccination, this being a little higher than that observed in Hong Kong and UK [24], [25], but much lower than that of the USA(66%) [26]. Chronic pulmonary disease (i.e. asthma), was the most common underlying medical condition. There are many studies to show that children hospitalized with influenza B are more likely to have an underlying risk than influenza A. But in present study, there were 15.0% children with influenza A and 12.1% children with influenza B having underlying risks. The possible reasons for the differences were: (1) lack of sensitivity of the immuno-fluorescent assay which might bias the results; (2)the relatively small sample size in our study might not be large enough to detect the differences between influenza A and B. (3) These may be the real condition in Suzhou China. Further prospective study is needed to test this hypothesis.

In other settings, children with ACIP indications were at increased risk for severe complications from influenza and hospitalization for influenza-related diseases [27], [28]. However, in our study, although the numbers are small, children with an ACIP indication for influenza vaccine had lower percentages of severe complications (such as septicemia, respiratory or heart failure, myocardial damage and hepatic injury) than those without ACIP indications. In addition, ACIP indication had no effect on the length of hospital stay in our study, which is contrary to the results of Keren [26] and Burton [29] that children with ACIP indications for influenza vaccination had longer hospital stays. However, children with ACIP indications for influenza vaccination had more hospitalization costs than that of children without ACIP indications.

Social and medical systems are unique in China, and more study is needed of the effect of these differences on medical care practice [30]. The 7.0 days median hospital stay in this study was much longer than that from USA (2 days) [31],Canada (5.3 days) [23] and that observed in other countries [25], [32]. A possible reason for this was the higher percentage of pneumonia cases seen in our study. In addition, with the one child policy in China, most parents pay more attention to their child, and tend to demand a longer hospital stay. Length of hospitalization of children ≤6 months old was longer than older children in our study. By contrast, Ampofo reported that [22], [32] children less than 6 months old had shorter hospitalization stay than older children. In older children it is possible that parents may be concerned regarding missed school days and hence they may prefer to have their children discharged sooner. We hope to evaluate these trends further in a prospective study. Aerosol or oxygen treatment was another factor influencing the length of hospitalization in our study.

Direct medical costs were large enough to be an important source of economic burden to families of children with influenza-related hospitalizations. Children with underlying chronic disease or other high risk factors had higher odds of having high-cost hospitalizations. The mean cost of hospitalization for a child hospitalized with influenza was US$624 in our study, which was similar with the Hong Kong study –HK$4200 (about US$541 with currently exchange rate: 1HK$ equal 0.1288 US$) per hospitalization in early 2000s [33], but much lower than the result from US–US$ 13159 per influenza hospitalization [26] and Hall [34] reported US$3055–38584 per child hospitalization with influenza infection. These differences are influenced by local economics and the differing medical care system, with the income and salary in China being much lower than that of US. We did not include the indirect cost of each hospitalization in our study. In addition, although all of the children had a chest X ray, most of them had the test prior to admission and therefore our hospitalization costs did not include this cost as this data was not available in our database. Our observed costs were much lower than a recently study in Hong Kong [35], which reported the mean direct and indirect cost of each child hospitalized was $1217.82 and $1328.33 for influenza A and B respectively.

During the pandemic period, there were special policies in China related to influenza management [36]. Although immuno-fluorescent assay did not specifically differentiate the 2009 pandemic H1N1 virus, we compared the influenza A cases during pandemic period and the seasonal influenza periods. The clinical characteristics of pandemic H1N1 cases were similar to the seasonal influenza A cases. The average hospitalization costs for the pandemic period children (US$ 696) were higher than that of seasonal influenza children (US$587) (data not shown). It seems the pandemic response policies had some impacts on the medical costs and care of the hospitalized children.

Some limitations to our study may have biased our results. Since this was a retrospective study, we relied upon influenza infection being diagnosed by an immuno-fluorescent method, which is less sensitive than RT-PCR for influenza virus testing. Additionally, we could not determine the subtypes of the influenza virus during the study period. Thus, we have begun a prospective study to further evaluate the burden of influenza in Suzhou, China using pcr testing.

### Conclusions

Influenza hospitalization in young children occurs throughout the year in Suzhou. Compared to other countries, in Suzhou influenza-related hospitalization have longer hospital stay and higher percentage of pneumonia. The direct medical cost of the influenza-associated hospitalization in children is described for the first time in Chinese medical system and is high relative to family income. Thus, effective strategies of influenza immunization of young children in China may beneficial.
